# First detection and full genomic analysis of Canine Circovirus in CPV-2 infected dogs in Colombia, South America

**DOI:** 10.1038/s41598-020-74630-8

**Published:** 2020-10-16

**Authors:** Sebastian Giraldo-Ramirez, Santiago Rendon-Marin, Diana S. Vargas-Bermudez, Jairo Jaime, Julian Ruiz-Saenz

**Affiliations:** 1 Grupo de Investigación en Ciencias Animales - GRICA, Facultad de Medicina Veterinaria Y Zootecnia, Universidad Cooperativa de Colombia, sede Bucaramanga, Calle 30A # 33-51, Bucaramanga, Colombia; 2 Departamento de Salud Animal, Centro de Investigación en Infectología E Inmunología Veterinaria (CI3V), Facultad de Medicina Veterinaria Y de Zootecnia, Universidad Nacional de Colombia, Sede Bogotá, Carrera 30 No. 45-03, CP 1100 Bogotá, Colombia

**Keywords:** Virology, Evolution

## Abstract

Canine Circovirus (CanineCV) is an emerging virus which since its first report in USA in 2012, it has been described worldwide. It was the second mammalian circovirus species identified in dogs and its role in canine enteritis is still being uncertain as much as its association in disease with the Canine Parvovirus-2 (CPV-2). Here, we aim to confirm for the first time the presence of CanineCV in Colombia and to develop phylogenetic evolutive analyses of CanineCV in CPV-2 positive animals. DNA from samples were extracted and PCR, full genome sequencing and phylogenetic analysis was performed to detect and characterize CanineCV. From a total of 30 CPV-2 positive samples, 16.6% (n = 5) were positives for CanineCV. Sequencing analysis of Colombian CanineCV wild-type strains displayed high identity to each other (99.5–99.7% nt; 99.7% aa). The full genome phylogenetic analysis confirmed that worldwide reported CanineCV strains were separated into four distinct genotypes in addition to a European origin of the South American CanineCV strains. This study demonstrated the importance of continue surveillance of emerging viruses in canine populations and confirm for the first time the circulation and origin of CanineCV in Colombia.

## Introduction

Canine Circovirus (CanineCV) is a non-enveloped, icosahedral virus with a single-stranded covalently closed circular DNA genome of 2 kb in length approximately, classified under the genus Circovirus within the family of *Circoviridae*^1^. CanineCV possesses an ambisense genomic organization with two majors inversely arranged ORFs encoding for the replicase and capsid proteins, respectively^2^. Until 2012, known mammalian circoviruses included the porcine circoviruses type 1 and type 2 (PCV1 and PCV2)^3^ and the worldwide identified bat circoviruses^4,5^. However, new circoviruses had been reported recently, such as the PCV3, which has been found in tissues or serum of healthy and pigs suffering from different clinical conditions^6^ and the CanineCV reported in serum samples of clinically healthy dogs in USA^1^.

Up to date, the role of CanineCV in canine enteritis and its clinical manifestations in dogs are still being uncertain. In Particular, dogs have been reported as infected by different Porcine circoviruses^7^. The PCV2 and PCV3 has been detected in dogs from Germany and China respectively^8,9^ and a recent epidemiological survey found the presence of 23,6% of PCV3 positive canine samples in the Guangxi Province, China^10^.

Some reports suggest that CanineCV might cause haemorrhagic diarrhoea in dogs. By PCR, CanineCV has been detected in the feces from 14 of 204 healthy dogs (6.9%) and 19 of 168 dogs with diarrhea (11.3%)^2^, and also, this agent has been detected in sporadic cases of dogs with vasculitis, histiocytic inflammation, or a combination^11,12^. On the other hand, the virus has been already reported in different wildlife carnivorous from Europe, mainly in Wolves (*Canis lupus*), red foxes (*Vulpes vulpes*) and virus from those wild animals share a genome identity up to 80%, suggesting that wildlife and dogs can interchange related circoviruses and that CanineCV could possibly be transmitted from dogs to wild canids, or vice versa^13–16^.

In pigs, experimental and natural disease studies have shown that PCV2 single infections hardly ever lead to severe clinical disease and that porcine circovirus associated diseases (PCVAD) are often accelerated or exacerbated by concurrent viral or bacterial infections^17–19^. However, porcine dermatitis and nephropathy syndrome (one of the PCVAD diseases) shares many of the histologic features of seen in CanineCV-positive dogs. Additionally, PCV2 has been reported to cause necrotizing lymphadenitis, vasculitis, or neurologic disease as similar as CanineCV and virus distribution in tissues is also similar between CanineCV and PCV2^2^. It is well known that gastrointestinal disorders in canines are one of the most common diseases reported in clinics of companion animal and that these disease can be caused by a number of synergistic viral, bacterial and parasitic pathogens^20^. Recent molecular surveys have proposed the role of CanineCV not only as a causative agent but also it might play a role as a co-pathogen in the development of gastrointestinal disorders, mainly acting in synergism with other enteric viruses such as Canine Parvovirus-2 (CPV-2)^20–23^.

In Colombia, CPV-2 has been previously related as main cause of gastrointestinal disease in CPV-2 non-vaccine and partial vaccinated animals^24^. Considering that CanineCV has been recently reported in other Latin-American countries^23,25^ and that it has never been reported in Colombia; the aim of this study was to confirm, for the first time, the presence of CanineCV in Colombia and to develop phylogenetic evolutive analyses of CanineCV in CPV-2 positive animals to understand the molecular epidemiology of this emerging virus in Colombia.

## Materials and methods

### Type of study, target population and sampling

A descriptive cross-sectional study was conducted with convenience sampling in canine patients with clinical signs of haemorrhagic diarrhoea and a presumptive diagnosis of parvovirus (gastrointestinal symptoms characteristic of parvovirus and/or bloody diarrhea) belonging to the Valley of Aburrá (Antioquia), Colombia. Approximately one gram of feces was collected from those patients and stored at − 80 °C until processing.

### DNA extraction

The QIAamp DNA Stool kit (Qiagen, Hilden, Germany) was used to extract viral DNA present in the stool samples following the manufacturer's recommendations. All samples were quantified using a NanoDrop 2000 (Thermo Fisher Scientific, Waltham, MA, USA) and RNA was stored at − 80 °C until used.

### PCR detection of CanineCV and CPV-2

For detection of CanineCV in dog samples, a conventional PCR assay was performed using a set of specific primers reported by Kotsias et al.^25^. Briefly, the primers Forward For-genomic-(5′-ATGGCTCAAGCTCAGGTTG-3′) and the Rev 533 Reverse primer (5′-CCGCACAGAACCTCCACTTC-3′) were used. To confirm CPV-2-positive samples, PCR amplification of VP2 was conducted using the Forward Ext1F primer (5′-ATGAGTGATGGAGCAGTTCA-3′) and the Ext3R Reverse primer (5′-AGGTGCTAGTTGAGATTTTTCATATAC-3′) described by Ref.^26^. In all cases, reactions were performed in a total volume of 25 ul containing 1 unit of Taq polymerase (Go taq flexi-Promega), 1 × Taq buffer, 2 µM MgCl_2_, 0.5 µM dNTPs, 20 pmol of each primer and 100 ng of extracted DNA. The PCR reactions were performed on a C1000 Touch BIORAD-DNA thermocycler (Biorad; CA, USA). Molecular grade water was used as a negative control for amplifications. PCR amplification results were visualized using 1.5% horizontal agarose gel electrophoresis stained with the Invitrogen SYBR Safe DNA Gel Stain (Thermo Fisher Scientific). The GeneRuler 100-bp DNA Plus Ladder (Thermo Fisher Scientific) was used as a molecular weight marker. Gels were visualized in the ultraviolet light Gel Doc XR + imaging system (Bio-Rad, Molecular imager, USA) by using ImageLab software.

### Amplification of CanineCV full genome, sequencing and sequence analysis

The CanineCV full genome of three dog samples were amplified using four sets of specific sequencing primers reported by Kotsias et al.^25^. Reactions were performed in a total volume of 25 ul containing 1 unit of AccuPrime Taq (Thermofisher), 1× AccuPrimer PCR Buffer I, 20 pmol of each primer and 50 ng of DNA extracted. The PCR reactions were performed on a C1000 Touch BIORAD-DNA thermocycler using a protocol consisting of denaturation at 94 °C for 2 min, followed by 35 cycles including a denaturation at 94 °C for 30 s, annealing at 56 °C for 30 s and extension at 68 °C for 1 min. PCR products were directly sequenced employing both directions at the commercial sequencing facility SSiGMol (Servicio de Secuenciación y Análisis Molecular, Instituto de Genética, Universidad Nacional de Colombia).

The chromatogram sequencing files were analyzed using Chromas v2.6 (Technelysium, Helensvale, Australia) and consensus contigs were assembled using SeqMan Pro platform in Lasergene Software v.15 (Lasergene INC. Madison, Wisconsin, USA). DNA sequences were aligned using ClustalW method and compared with the nucleotide sequences from CanineCV from domestic dogs, wildlife and other reference circoviruses from GenBank. The best-fit model of nucleotide substitution was selected (GTR + G + I) and Maximum-likelihood and neighbor joining trees with a bootstrap value of 1000 were inferred. Phylogenetic analysis was inferred as previously reported, in order to characterize the CanineCV into four distinct genotypes (denoted as CanineCV-1 to -4)^21^. All Phylogenetic analysis were performed by using MEGA 7.0 for Windows. The complete genome sequences of Colombian CanineCV has been deposited in GenBank under the accession numbers [MT293519 to MT293521]. Recombination events were evaluated using RDP4.0 and Simplot v3.5.1 subprograms.

### Amino acid analysis of the CanineCV

The deduced amino acid sequences Colombian CanineCV where aligned with cognate circoviruses protein sequences from GenBank using MEGA 7 to explore their amino acid profile and the potential differences among wild-type and domestic strains worldwide. The nucleotide and amino acid differences were assessed as uncorrected (p) distances. Prediction of potential N-linked glycosylation sites was performed with NetNGlyc 1.0^27^. Moreover, a non-neutral selection assay was carried out using FUBRAR from Datamonkey 2.0^28,29^.

### CanineCV timeline of evolution

The mean substitution rate (substitutions per site per year), the time to the most recent common ancestor (tMRCA), the geographic origin, and the overall spatial dynamics of the major CanineCV clades were inferred using the Bayesian approach of the Markov Chain Monte Carlo implemented in the BEAUti/BEAST v1.8.4 package^30^. The analysis was accomplished employing a strict molecular clock with a constant population size, and 3E07 generations were run in order to ensure an effective population size greater than 200 for the evaluated parameters using the Tracer v1.7 program^30^. The initial 10% of the MCMC, which corresponds with low probability states at the beginning of the chain, was eliminated. The tree of maximum credibility of the Maximum Clade Credibility clades was built with TreeAnnotator and visualized with FigTree v1.4.3.

### Ethical considerations

This study was approved by the Ethics Committee for Animal Experimentation of the Cooperative University of Colombia in Bucaramanga. All experiments were performed in accordance with relevant guidelines and regulations. Dog owners signed informed consent forms approved by the ethics committee.

## Results

From a total of 30 CPV-2 positive samples, 16.6% (n = 5) were positives for CanineCV. Three samples belong to the Antioquia Region and two of them to the Bogotá area. Although five positive samples were detected, only three complete genome sequences, including the reported 2063 bp and two partial CanineCV fragments were obtained. Table 1 summarizes age, gender, breed, vaccination status, clinical outcome and NCBI accession numbers.

**Table 1:** Information on CanineCV-positive samples included in the present study.

Sample	CPV-2 variant	Age (months)	Sex	CPV-2 Vaccination status	NCBI
MDE-1	CPV-2a	2	Female	Up to date	MT293519
MDE-2	CPV-2a	2	Female	Up to date	MT293520
MDE-3	CPV-2a	2	Male	Up to date	MT293521

An initial BLASTn search revealed that Colombian wild-type viruses showed 97.53% maximum nucleotide identity to Canine circovirus strain Bari/411-13 (GenBank accession number KJ530972.1) that belongs to a strain associated with fatal hemorrhagic enteritis in dog in Italy; also, it has a 97.04% nucleotide identity to a strain isolated from a dog from California (USA) reported with severe hemorrhagic gastroenteritis, vasculitis, and granulomatous lymphadenitis (KC241984) and 96.88% nucleotide identity to a German strain (Ha13 strain-KF887949). The geographically closest sequence was to a CanineCV isolated from an outbreak of fatal gastroenteritis of dogs in Argentina (MK033608). The three Colombian wild-type strains submitted to analysis displayed high identity to each other (99.5–99.7% nt; 99.7% aa).

**Figure 1:**
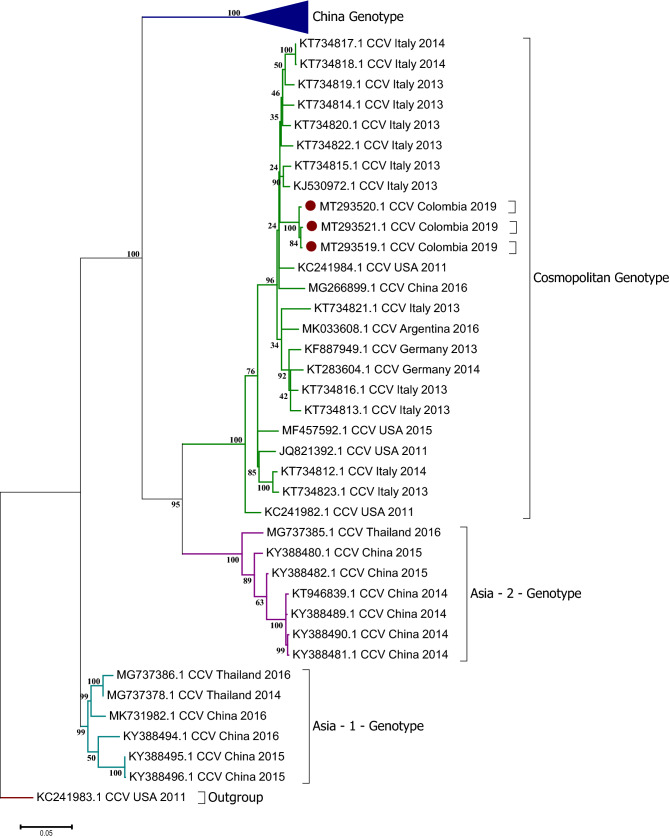
Maximum Likelihood tree based on full genome CanineCV sequences. The tree with the highest log likelihood is shown. The tree is drawn to scale, with branch lengths measured in the number of substitutions per site. The analysis involved 78 nucleotide sequences and a total of 2063 positions in the final dataset. Red dots indicate Colombian strains. Phylogenetic analysis was performed by using MEGA 7.0 for Windows.

The phylogenetic relationships based on the nucleotide alignment of complete genome sequences inferred by distances (neighbor joining) and characters (maximum likelihood) approaches exhibited trees with the same topology (data no shown). As previously reported^21^, we confirmed that worldwide reported CanineCV strains were separated into four distinct genotypes (previously denoted as CanineCV-1 to -4) with a greater majority of sequences with a Chinese origin and a paraphyletic distribution of each subclade. Due to the current distribution of new CanineCV sequences, we propose to designate different clades according to the geographical distribution (Genotypes). In this order, “Genotype China” only include Chinese sequences, Genotypes Asia-1 and Asia-2 includes sequences from China and Thailand and the widely distributed clade named as “Cosmopolitan Genotype” (previously denoted as CanineCV-1 genotype), due to the presence of sequences from different continents as Europe (Italy and Germany), Asia (China), North America (USA) and South America (Argentina and Colombia) (Fig. 1).

**Figure 2:**
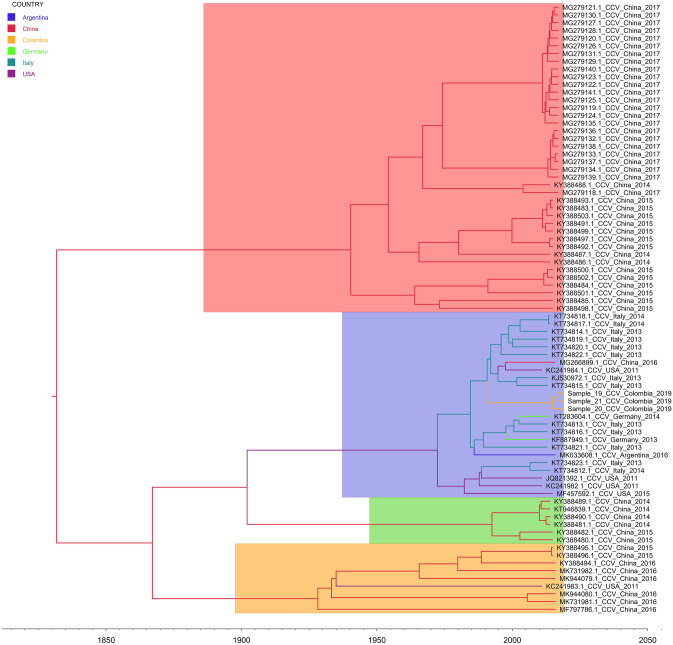
Phylogenetic evolutionary tree of Colombian CanineCV. The tree was generated using full genome sequences from different countries. The sequences are identified with the accession number, country and date of collection. The timeline shows the moment of evolutionary divergence of the sequences of each country. Different genotypes are highlighted: Red- China genotype, Blue: Cosmopolitan genotype, Green: Asia-I genotype, Yellow: Asia-II genotype. Maximum credibility tree of the MCMC was built with TreeAnnotator and visualized with FigTree v1.4.3.

The mean estimated tMRCA for the full CanineCV genome was 1888 (HDP 95% Interval 1813-1942). The phylogeographic analysis supported the Chinese Origin of the CanineCV and a posterior segregation into four subclades being the CanineCV-1 the globally distributed clade (Fig. 2—Cosmopolitan clade). The worldwide distribution of this subclade showed a probable monophyletic Chinese origin and a following dissemination to North America and Europe. It is important to highlight that although Argentinian and Colombian strains have different time origins, both come from Italian strains. This same pattern could be observed for the German strains.

Analysis of the deduced amino acid sequences of the full-length Colombian CanineCV showed the presence of previously reported substitutions in amino acids E194D, M252I and H681Q. Also, we found the presence of a unique mutation in the capsid protein (I371F) which is present in all Colombian strains sequence. No recombination events were obtained in the circulating CanineCv from Colombia, however previously reported recombination events were found^31^.

N-Glycosylation sites for CanineCV capsid protein have not been reported yet. However, a single putative N-glycosylation site of PCV2 capsid protein was described at position N143YS^32^. For all the CanineCV Colombian strains, we identified a single potential glycosylation site at position sequence N134AT. N-glycosylation potential sites was evaluated for the replicase protein; however, no N-glycosylation were found for this protein.

Furthermore, we assessed non-neutral selection employing FUBAR method from Data Monkey. For the replicase protein, we found that the CanineCV sequences included four sites under positive selection: 10, 168, 265 and 269, which had a posterior probability of 0.9 with Bayes factor as 1572.313, 60.105, 39.932 and 113.960, respectively. We also observed 178 sites under negative selection which had a posterior probability of 0.9 with Bayes factor < 1. For the capsid protein, we found one site under positive selection: 16 with a posterior probability of 0.9 and Bayes factor of 58.161. Moreover, we also identify 184 sites under negative selection with a posterior probability of 0.9 and Bayes factor < 1.

## Discussion

CanineCV has become as an important emerging virus in different part of the word. In the present study, CPV-2 positive dog samples from Colombia with an acute enteritis disease^33^ were subjected to additional CanineCV analyses. Our results confirm the circulation of CanineCV in Colombian dogs for the first time in co-infection with CPV-2. Based on this epidemiological issue, our results confirmed the wide viral circulation in all south American continent. The CanineCV was first described in Brazil in 1.34% of healthy dog serum samples in a canine virome study^23^. Later on, the virus was reported in Argentina in an outbreak of fatal gastroenteritis in dogs^25^, which was detected in multiple organs of the infected animals (pancreas, liver, kidney, lung, lymph nodes, spleen and large intestine).

Since Kapoor et al., reported the detection of CanineCV for the first time in the serum of healthy dogs in the USA^1^, several studies have demonstrated the high circulation of CanineCV in different canine populations around world, with prevalences (or diagnostic rates) from 1 to 33% of the evaluated samples and it has been reported in almost all continents^20,21,23^. Our results strongly agree with the frequencies reported for northern China where the prevalence of CanineCV among diarrheic dogs was 15.6%^21^.

The critical role of CanineCV as single diarrheagenic agent has been extensively discussed and its pathogenicity has not been clarified yet. The CanineCV has been detected in both symptomatic and asymptomatic dogs^21,25,34^, and only vasculitis and histiocytic inflammation have been associated in some dogs^2^. CanineCV have not been involved as the primary causative agent of the acute haemorrhagic diarrhoea syndrome in dogs, but might play an important role as a negative co-factor in the disease outcome in dogs with CPV-2 infection^34^.

Currently, some authors has recently proposed a synergistic effect of CanineCV and CPV-2 co infection that allowed development of clinical disease in dogs despite up-to-date vaccination against CPV-2^22^. Although, with very few patients, our study suggests the same hypothesis due to all animals enrolled in this study has updated vaccination schedule for CPV-2 and developed clinical signs and also all of them were positives for CPV-2 and CanineCV (Table 1). It is important to note that synergistic relationship between circoviruses and parvoviruses was first reported in pigs.

In pigs, PCV2 has been associated with different disease syndromes known as PCVAD, which are worsened by coinfection with PPV and other pathogens^17,35,36^. In the case of natural and experimental coinfection with PCV2 and PPV, they have been shown to participate in the manifestation of the reproductive complex stillbirth, mummification, embryonic death, and infertility (SMEDI)^37^, mainly in the first half of gestation and after day 70 the birth of healthy and immunocompetent piglets^38^. This same coinfection in some cases has been associated with other types of clinical presentations in pigs where the synergism of these pathogens has not been established with precision, but it is proposed that the tropism of PCV2 by cells of the immune system favors the development of different syndromes^39^. Pigs inoculated with PCV2 and PPV exhibited significantly higher TNF-α levels compared to pigs inoculated with PCV2 or PPV alone. TNF-α levels in sera from infected animals are inversely correlated with body weight of pigs that have been experimentally infected with PCV2 and PPV, suggesting that PPV is associated with excessive production of TNF-α. in PMWS induced by PCV2^40^ Furthermore, for PCV2 it is well known that to induce diseases by porcine circovirus or PCVAD there is a direct relationship between the viral load of infected pigs and the development of the disease^41^.

Our phylogenetic analysis confirmed that most of the sequences that have been reported to date in GenBank, belongs to the Asia and China Genotypes (Fig. 1) and that a strong phylogeographic patterns could be observed in at least three of the four globally reported genotypes. This geographic pattern has been described for other canine viruses such as the Canine Distemper Virus (CDV), where global evolutionary history of current CDV strains has been tracked showing that ancestral strains had evolve in independent model on each geographic regions from a single ancestral geographic origin^42,43^.

On the other hand, the existence of an intercontinental or cosmopolitan genotype with a single geographic origin (Fig. 2), demonstrates the susceptibility for the transmission of infectious diseases between canine populations in a globalized world. It is important to highlight that CanineCV transmission from Europe (Italy) to South America coincides with the second migration wave to Ecuador of the Europe I clade of CPV-2 from Italy at early 2000 decade^44^. This data enables us to propose that viral migrations occurs in co-infection with CPV-2 exhibiting the importance of the study of more sequences form dog samples to confirm whether that extensive migrations and local differentiation have been a driving force for CanineCV evolution in South America in the recent past as it has been stated for the CPV-2^44,45^. This evolution mark can be explained by the existence of specific amino acid changes (I371F) in Colombian CanineCV sequences which indicates that some viruses were undergoing diversification that may be related to local selection pressures and adaptation^13,44^, a fact that need to be supported in the near future by analyzing bigger datasets in different times and geographic locations and that it finally needs to be evaluated in the context of its impact on viral pathogenesis.

It has been stablished for other relevant canine viruses such as CPV-2 and CDV that multiple viral migrations from Europe to South America^44,46,47^. The same pattern could be seen for the CanineCV, for which there are at least two migrations events from Italy, one to Colombia and later, another to Argentina (Fig. 2). Recently, the circulation of CanineCV in Brazil was described^23^, although the sequences of this study could not be included in our analysis because they are partial sequences, the analysis make possible to assume that the Brazilian strains also share origin with Italian sequences supporting the Italian migration route of the CanineCV to South America^23^.

Circoviruses that belong to the same species share more than 75% and 70% of nucleotide identity in the complete genome and capsid protein encoding gene, respectively^3^, Based on this criterion, it has been proposed the existence of a unique carnivore circovirus species that includes CanineCV detected in dogs and wildlife, mainly in foxes^13^. Our phylogenetic and evolutionary analyzes, including a Fox circovirus sequence (KP260925), enable us to propose a common Asian origin of wild and domestic carnivorous circoviruses (Figs. 1 and 2). This observation allow us to suggest that it is important to enhance surveillance in wild populations closely found to domestic places due to an analysis of CanineCV published recently in Italian red foxes which has shown the presence of a closely related CanineCV from dogs (*Canis lupus familiaris*) rather than those from foxes naturally infecting wild populations, suggesting a possible transmission between the two species^48^.

The N-glycosylation have been involved in the proper folding of a vast array of surface viral protein which might pay a crucial role in pathogenesis and host immune response, due to the presence of this post-transcriptional modification imply antigenic conformations of viral capsid^49^. Our study showed a conserved N-glycosylation site among all the CanineCV Colombian strains, with the same potential motif. As it has not been reported for CanineCV, the importance of a single N-glycosylation site of PCV2 has been demonstrated in vitro, and its implications in the immune evasion, located in the same region of the CanineCV potential site, at position N143YS^32^. These results suggest that N-glycosylation sites could be important in the capsid protein for antibodies recognition and vaccines development in the context of Circoviruses. Some structural analysis could be accomplished to establish whether this N-glycosylation has an important role in the aspects mentioned above.

Regarding the selection analysis, it has not been reported neither positive nor negative selection sites for CanineCV yet. However, employing FUBAR method, which assumes that selection pressure for each site must be constant throughout the phylogenetic reconstruction, we identify sites under positive selection for both the replicase and capsid proteins. We found four sites for the replicase protein and one for the capsid protein. Some studies might be carried out to set up whether these sites are related to pathogenicity aspects considering the amount of positive selection sites of replicase protein, and immune evasion or antibodies recognition, as reported previously for other canine viral pathogens^46,47^.

In conclusion, after its first report in the USA, the CanineCV has proved to be a virus of worldwide distribution, and probably, an emerging virus in different canine populations around the world (domestic and wild). Our results allowed us to confirm the wide distribution of the CanineCV in the Americas and confirmed the circulation of the CanineCV in Colombia for the first time. Additionally, our results showed the presence of three genotypes with geographic restriction for the Asian continent and the cosmopolitan circulation of a single viral genotype, which has been distributed from Europe to a vast array of regions around world, including South America.

It is necessary to increase the surveillance of this agent in domestic populations to better understand its role as a pathogenic agent and define its role as a required and a concomitant agent in co-infection with CPV-2 for the development of clinical disease even in vaccinated animals. Finally, we call on to the international animal health professionals to combine research efforts that enable us to understand the multiple knowledge gaps (pathogenesis, distribution, ecoepidemiology, among others) that are around this new agent that exposes multiple canine populations at risks.

## Data Availability

The datasets generated during and/or analyzed during the current study are available from the corresponding author upon request.
